# Synthesis, characterization, and drug release properties of enzyme-responsive oxamide-bridged mesoporous organosilica nanoparticles

**DOI:** 10.55730/1300-0527.3742

**Published:** 2025-04-08

**Authors:** Yaşar GÖK, Halil Zeki GÖK, Osman Tayyar ARLI, Tuğçe TÜRKASLAN

**Affiliations:** Department of Chemistry, Faculty of Science and Letters, Burdur Mehmet Akif Ersoy University, Burdur, Turkiye

**Keywords:** Drug delivery system, enzyme responsive, mesoporous organosilica, drug release

## Abstract

In this study, we report a novel enzyme-responsive mesoporous organosilica nanocarrier (MON) and its drug delivery properties in the presence of the trypsin enzyme. The mesoporous organosilica nanocarrier was synthesized by using the oxamide bridged bissilyl compound (2) and 1,4-bis(triethoxysilyl)benzene (BTSB). To compare the enzyme responsiveness of MON, the reference periodic mesoporous organosilica (PMO) nanocarrier was also synthesized using BTSB only. The structural determinations of the synthesized organic compounds were carried out by ^1^H, ^13^C-NMR, FT-IR, and MS, while FT-IR, XRD, SEM, EDS, transmission electron microscopy (TEM), TGA, and N_2_ sorption analyses were used for the characterization of MON and PMO. Drug loading and release properties of both nanocarriers were studied by using curcumin as a model drug. The loading capacities were determined as 11.4% for MON and 2.9% for PMO. The enzyme-responsive drug release of the drug-loaded nanocarrier curcumin-loaded MON (Cur@MON) was investigated in the presence and absence of the trypsin enzyme at physiological pH. The results showed that the oxamide structure undergoes selective cleavage of amide bonds in the presence of the trypsin enzyme, causing the nanocarrier to release curcumin.

## 1. Introduction

Over the last decade, the number of studies on novel and powerful drug delivery systems has been increasing rapidly. New approaches and methods are being developed, especially on the controlled release of drugs [1–6]. To date, different systems such as liposomes [7,8], polymers [9,10], polymerosomes [11], silica nanoparticles [12–14] and hydrogels, [15,16] have been used with different formulations as drug delivery systems. Some of these systems are also approved by the American Food and Drug Administration (FDA) [17]. However, serious problems may arise in the long-term use of some of these systems. For example, dose-dependent toxicity, drug leakage, stability, and biocompatibility are some of the problems encountered [18]. Apart from these problems, the chemical and physical compatibility between the drug to be loaded and the drug delivery system is also very important [19]. When selecting or designing nanocarriers to be used as carriers for a particular type of drug, it is critical to know the properties and behavior of the drug to achieve an optimal encapsulation efficiency and desired release profile [6, 20–22]. Taking into account the compatibility between the drug and the drug carrier, and especially the behavior of the drug in the biological system, ensuring that the drug is released as desired can provide solutions to the problems encountered in developing a new drug delivery system. In addition, the interaction of the biological system with the drug-drug carrier also guides the design of new systems. For example, adding different trigger mechanisms to drug carrier systems is one of the effective ways to prevent premature leakage and achieve controlled drug release [23–26]. Triggers sensitive to physical, chemical or biological changes are commonly used as trigger mechanisms in the design of the new drug delivery systems to ensure optimal drug release [27–29].

Considering these important features that must be taken into account in drug delivery systems, mesoporous silica nanoparticles (MSNs) may be ideal candidates. The reason for this is that MSNs are more resistant to degradation and mechanical stress compared to other nanocarriers due to the strong Si–O bonds they contain [30]. Furthermore, the FDA reported that taking silicates up to 1500 mg per day does not harm human health [31]. In addition, drugs loaded on silica-based nanocarriers can be transported by the carrier without leaking and can be released with a trigger at the desired time [32,33]. Although intensive studies have been carried out by researchers on controlled drug release and trigger-sensitive silica-based drug delivery systems [34,35], no study was conducted on enzyme-responsive silica-based drug transport systems triggered by biological stimuli until 2015 [36], despite the abundance of studies using chemical and physical stimuli as triggers.

Enzymes perform very important functions in our body. For instance, enzymes can bind covalently to polymer chains or can specifically break, cleave, or open certain chemical bonds [37]. In 2015, Fatieiev and his colleagues synthesized the first enzyme-responsive PMO nanocarrier system by taking advantage of this feature of enzymes and tested it as a drug delivery system [36]. However, they reported that the porosity of the mesoporous structure obtained in this study was very low, with a value of approximately 25 m^2^/g. In 2016, Maggini et al. synthesized a PMO nanocarrier based on an enzyme-responsive lysine bridged bissilyl compound and tested this structure for the transport of the anticancer drug doxorubicin (DOX) [38]. In the study, DOX could only be loaded into the nanocarrier at a rate of 2%. In both studies, only the bridged-organobissilyl compound was used as the silane source in the synthesis of PMOs. As a result of these studies, it was concluded that amorphous and aggregated nanocarriers can be obtained, and that intramolecular H-bonds significantly narrow the pore entrances. After the undesired results obtained in the enzyme-responsive PMO synthesis by Fatieiev and his colleagues in 2015, Croissant and his colleagues repeated the synthesis by adding a second silane compound alongside the bridged organosilane compound, unlike the synthesis route used by Fatieiev and his team [39]. After this experiment, it was determined that the MON obtained had a larger surface area and formed fewer hydrogen bonds. In their drug loading studies, they managed to load up to 84% of the drug. Additionally, drug release from the MON structure could be achieved in an enzyme-controlled manner.

Considering these studies, a new enzyme-responsive MON was synthesized, characterized, loaded with curcumin, and the drug release properties of this nanocarrier were examined in the presence and absence of the trypsin enzyme. The MON structure was synthesized by the cocondensation reaction of 1,4-bis(triethoxysilyl)benzene (BTSB) and the new oxamide-bridged bissilyl compound (2). This synthesis approach allowed the homogeneous distribution of oxamide functional groups on the nanoparticle. Enzyme-responsive drug release studies have shown that the oxamide structure undergoes selective cleavage of amide bonds in the presence of trypsin, causing the nanocarrier to release curcumin.

## 2. Materials and methods

### 2.1. Materials

2-((3-(triethoxysilyl)propyl)disulfanyl)aniline (1) and the reference PMO were synthesized by applying the methods in the literature [40,41]. All chemicals, except curcumin, were purchased from Merck KGaA (Darmstadt, Germany). Curcumin was obtained from ABCR GmbH (Karlsruhe, Germany). All solvents used during the syntheses were purified according to the methods specified by Perrin and Armarego [42]. For other processes, technical-grade solvents were used.

### 2.2. Characterization techniques

Characterization studies were performed according to techniques previously published by our group [40]. Fourier transform infrared (FT-IR) spectroscopy was performed using the KBr pellet technique (Shimadzu IR Affinity-1S, Shimadzu Corp., Kyoto, Japan). Nuclear magnetic resonance (NMR) measurements for ^1^H and ^13^C were acquired in CDCl_3_ using a Bruker AVANCE III 400 MHz NaNoBay spectrometer (Bruker BioSpin GmbH, Rheinstetten, Germany). Mass spectrometry (MS) measurements were recorded using the matrix-assisted laser desorption/ionization time-of-flight (MALDI-TOF) technique (Bruker Daltonics GmbH & Co. KG, Bremen, Germany). X-ray diffraction (XRD) measurements were performed using Cu-Kα radiation in the wide-angle range (2θ = 5°–80°) with a PANalytical Empyrean diffractometer (Malvern PANalytical B. V., Almelo, Netherlands). Nitrogen sorption isotherms were recorded using a Micromeritics Tristar II surface area and porosity analyzer (Micromeritics Instrument Corp., Norcross, GA, USA) for determining the specific surface area and pore size distribution. The Brunauer–Emmett–Teller (BET) method was used to calculate the surface area of the mesoporous materials, and the Barrett, Joyner, and Halenda (BJH) method was used to calculate the pore size distribution. The morphology and elemental composition of the samples were examined by scanning electron microscopy and energy-dispersive spectroscopy (SEM, EDS) using a JEOL JEM-1400 PLUS instrument (JEOL Ltd., Tokyo, Japan). Thermogravimetric analysis (TGA) measurements were performed using a Seiko SII TG/DTA 7200 instrument (Seiko Instruments Inc., Chiba, Japan) with a flow rate of 2 mL min^−1^ and a heating rate of 10 °C min^−1^ under N_2_ atmosphere. A UV-Vis spectrophotometer (SP-3000 Nano, Optima, Tokyo, Japan) was used for the measurements in drug loading and release experiments.

### 2.3. Preparation of organic and inorganic materials

#### 2.3.1. Synthesis of N_1_N_2_-bis(2-((3-(triethoxysilyl)propyl)thio)phenyl)oxalamide (2)

30 mL of dry dichloromethane and 2-((3-(triethoxysilyl)propyl)disulfanyl)aniline (1) (1 g, 3.36 mmol) were added to the cryostat cell at 0 °C and stirred for 15 min under an inert atmosphere. At this temperature, the solution of oxalyl chloride (0.32 g, 2.35 mmol) in dry dichloromethane was added dropwise to the reaction medium with the help of a dropping funnel. After the addition was completed, the reaction content was stirred at room temperature for 1 h. The progress of the reaction was monitored by TLC using the hexane–ethyl acetate (8:2) solvent system. One hour later, the solvent of the reaction was removed under reduced pressure. The crude product was stirred with the hexane–ethyl acetate (8:2) solvent system for 1 h. The mixture was filtered and the filtrate was dried under vacuum. The targeted product (2) was obtained as brown oil (yield: 1 g, 42%). FT-IR (ATR), ν (cm^−1^): 3290, 3053, 2970, 2920, 1745, 1707, 1686, 1575, 1504, 1433, 1298, 1157, 1064, 1022, 964, and 754. Proton nuclear magnetic resonance (^1^H NMR) (400 MHz, CDCl_3_, *d*, ppm): 0.69 (4H, t, *J* = 8.5 Hz, CH_2_Si), 1.06 (18H, t, *J* = 6.8 Hz, OCH_2_CH_3_), 1.52 (4H, p, *J* = 7.8 Hz, CH_2_CH_2_Si), 2.90 (4H, t, *J* = 7.3 Hz, CH_2_CH_2_CH_2_Si), 3.65 (12H, q, *J* = 7.0 Hz, OCH_2_CH_3_), 7.24 (2H, dt, *J* = 7.8, 1.3 Hz, Ph-H), 7.42 (2H, dt, *J* = 7.8, 1.3 Hz, Ph-H), 7.62 (2H, dt, *J* = 8.0, 1.3 Hz, Ph-H), 8.24 (2H, dd, *J* = 8.0, 1.3 Hz, Ph-H), 10.58 (2H, s, NH). Carbon-13 nuclear magnetic resonance (^13^C NMR) (100 MHz, CDCl_3_, *d*:, ppm): 157.29 (C), 137.29 (C), 134.07 (CH), 128.84 (CH), 125.72 (CH), 125.40 (C), 120.34 (CH), 57.65 (CH_2_), 37.74 (CH_2_), 22.47 (CH_2_), 18.07 (CH_3_), and 8.98 (CH_2_). MS (MALDI-TOF): calculated [M]^+^ for C_32_H_52_N_2_O_8_S_2_Si_2_: 712.27 found: 733.65 [M + H_2_O + H] ^+^; 749.53 [M + 2H_2_O] ^+^ (See Supplementary Figures S1–S3).

#### 2.3.2. Preparation of mesoporous organosilica material (MON)

Cetyltrimethylammonium bromide (CTAB) (0.76 g, 2.08 mmol) and 363 mL of ultrapure water were added to a three-necked flask and stirred at 40 °C until CTAB dissolved. Then, the temperature was increased to 80 °C by adding 2.7 mL of 2 M NaOH solution and stirring for another 1 h. Then, BTSB (2.8 g, 7 mmol, 2.5 mL) and the oxamide-bridged bissilyl compound (2) were dissolved in 2.5 mL of acetone (0.51 g, 0.70 mmol) and added to the reaction medium. After stirring for another 2 h at 80 °C, the temperature was turned off and stirring was continued. The reaction content was cooled to room temperature and then filtered. The remaining solid part was washed first with distilled water, then with ethyl alcohol, and dried under vacuum. In order to remove CTAB from the structure of the obtained substance, CTAB@MON, sonication was performed twice for 30 min at 45 °C with an ethanol solution of NH_4_NO_3_ (6 g/L). After sonication, the solid was filtered, washed three times with ethyl alcohol, once with water, and again with alcohol. The resulting solid was dried under vacuum.

#### 2.3.3. Preparation of reference periodic mesoporous organosilica material (PMO)

For the synthesis of PMO, the method used in MON synthesis was followed. Unlike MON, 1,4-bis(triethoxysilyl)benzene (BTSB) was used as the sole silane source in the synthesis of PMO, without the use of the oxamide-bridged bissilyl compound (2) [43].

### 2.4. Curcumin loading and release

#### 2.4.1. Curcumin loading

Curcumin loading onto MON and PMO nanocarriers was performed according to our previously published method [43]. To prepare the 4 mg/mL curcumin ethanol stock solution, 400 mg of curcumin was added to a 100 mL volumetric flask, ethanol was added up to the 100 mL line of the flask, and the solution was stirred until complete dissolution at room temperature. Working solutions of 20, 10, 5, 2.5, 1.25, and 0.625 μg/mL were prepared using this stock solution. Then, a calibration graph was created for curcumin at 420 nm (see Supplementary Figures S4–S5). In order to load curcumin into MON and PMO, 25 mL of curcumin stock solution and 100 mg of MON or PMO were put together and sonicated. The obtained solutions were mixed at room temperature and in the dark for 24 h, and then centrifuged to separate MON and PMO from the curcumin loading solutions.

The remaining solid fractions were washed with 20 mL of ethanol and centrifuged. The drying process of the solid part was carried out in a vacuum oven. The absorbance of the combined loading and washing solutions was read at λ = 420 nm, and the curcumin concentration corresponding to this value was found using the standard calibration curve. Curcumin loading capacity was calculated using Eq. 1 [40,43,44]. The amount of curcumin in nanocarriers was also confirmed by TGA analysis.


(1) $$Loading\;capacity\;(\%)=\frac{the\;loaded\;amount\;of\;curcumin\;(mg)}{the\;loaded\;amount\;of\;curcumin(mg)+the\;amount\;of\;MON\;(mg)}\times 100$$

#### 2.4.2. Curcumin release

pH effect: The methods in the literature were followed for drug release studies [45,46]. Briefly, curcumin-loaded nanocarriers Cur@MON and Cur@PMO were added to 10 mL of phosphate-buffered saline (PBS) containing 10% (v/v) Tween-80 in a test tube and mixed at 37 °C and 100 rpm. Samples were taken from the release medium at regular intervals and centrifuged, and the absorbance of the supernatant was read at 420 nm. The amount of curcumin corresponding to the reading was calculated using the standard calibration curve.

Enzyme (trypsin) effect: The curcumin release study in the presence of trypsin enzyme was performed according to the method given in the literature [39]. Curcumin-loaded Cur@MON and Cur@PMO were added to 2 mL of PBS buffer (pH = 7.4) containing 10% (v/v) Tween-80 and dispersed using a sonicator. Then, trypsin (25%, 2 mL) was added and mixed at 100 rpm at 37 °C. Samples were taken from the release medium at regular intervals and centrifuged, and the absorbance of the supernatant was read at 420 nm. The amount of curcumin corresponding to the reading was calculated using the standard calibration curve.

## 3. Results and discussion

### 3.1. Characterization

**Figure 1:**
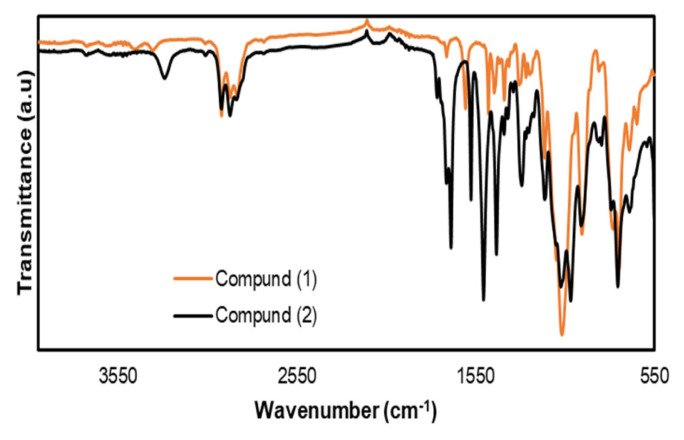
FT-IR spectra of (1) and (2).

**Scheme 1:**
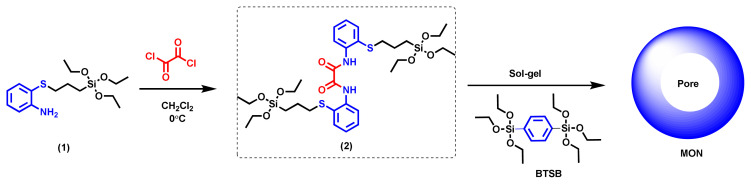
Synthesis of the mesoporous organosilica nanocarrier MON.

The synthesis pathway followed for the enzyme-responsive nanocarrier MON is given in Scheme 1. To obtain the trypsin-sensitive oxamide-bridged bissilyl compound (2), the synthesis of compound (1) was first performed according to the method given in the literature [40]. The oxamide-bridged alkoxysilane compound (2) was synthesized by reacting compound (1) with oxalyl chloride in dry dichloromethane. After applying the necessary purification processes to the obtained crude product, the target compound (2) was obtained with a yield of 42%. Characterization of compound (2) was carried out using ^1^H-NMR, ^13^C-NMR, FT-IR, and MS analysis. The FT-IR spectrum of compound (2) is shown in Figure 1. The stretching vibration observed at 3290 cm^−1^ in the FT-IR spectrum of (2) is due to the addition of oxalyl chloride to the primary amine (-NH_2_) group in compound (1) [47,48]. This observed value clearly shows that the primary amine was transformed into a secondary amine. This transformation was also clearly observed in the ^1^H-NMR spectrum. The -NH_2_ group observed at δ = 4.33 ppm in the ^1^H-NMR spectrum of compound (1) [40] disappeared in the ^1^H-NMR spectrum of (2), and a new signal was observed as a singlet in the downfield region at δ = 10.56 ppm due to the electron-withdrawing effect of the C=O group in the amide structure after the addition of oxalyl chloride to (1) (Figure S1). The stretching vibration at 1686 cm^−1^ in the FT-IR spectrum of (2) and the carbon resonance at δ = 157.29 ppm in the ^13^C-NMR spectrum clearly indicate the presence of the C=O group in the structure of (2) (Figure S2). Finally, in the mass spectrum of (2) taken with the MALDI-TOF technique, the molecular ion peaks observed at m/z = 733.65 [M + H_2_O + H]^+^ and 749.53 [M + 2H_2_O]^+^ support the proposed structure for (2) (Figure S3).

**Figure 2:**
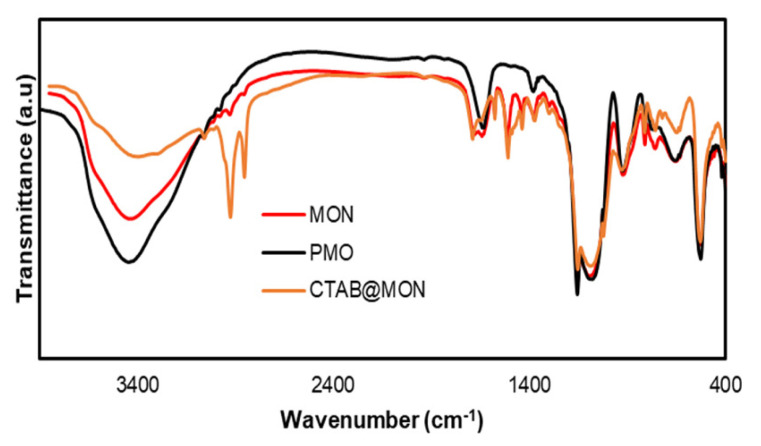
FT-IR spectra of MON, PMO, and CTAB@MON.

After elucidating the structure of compound (2), the nanocarriers MON and PMO were synthesized using the sol-gel method (Scheme 1). The reference nanoparticle PMO was also synthesized by repeating the synthesis method given for MON using only BTSB. When the FT-IR spectrum of MON is examined in Figure 2, the stretching vibrations observed at 2920 and 2850 cm^−1^ show that CTAB is present in the pores. Similar signals were also observed in the FT-IR spectrum of PMO. After sonication of MON and PMO nanocarrier dispersions in the ammonium nitrate in ethyl alcohol solution, the stretching vibrations at 2920 and 2850 cm^−1^ disappeared in the FT-IR spectra of the MON and PMO nanocarriers, indicating the successful removal of CTAB from the structures. The observation of vibration bands at 3059 and 3010 cm^−1^ indicates the presence of the aromatic ring in the MON structure, and vibrations for the propyloxamide groups appeared at 2972, 2924, and 2846 cm^−1^, as expected. The amide group was characterized by an intense stretching vibration at 1653 cm^−1^ for the C=O group and a bending vibration at 1510 cm^−1^ for the -NH group. Vibration signals belonging to the Si-O-Si group in the siloxane network were observed at 1143 and 1074 cm^−1^ [39,49,50]. When the FT-IR spectrum of PMO was examined, it was noted that it was very similar to the MON spectrum, except that the signals belonging to the propyloxamide group were not observed.

**Figure 3:**
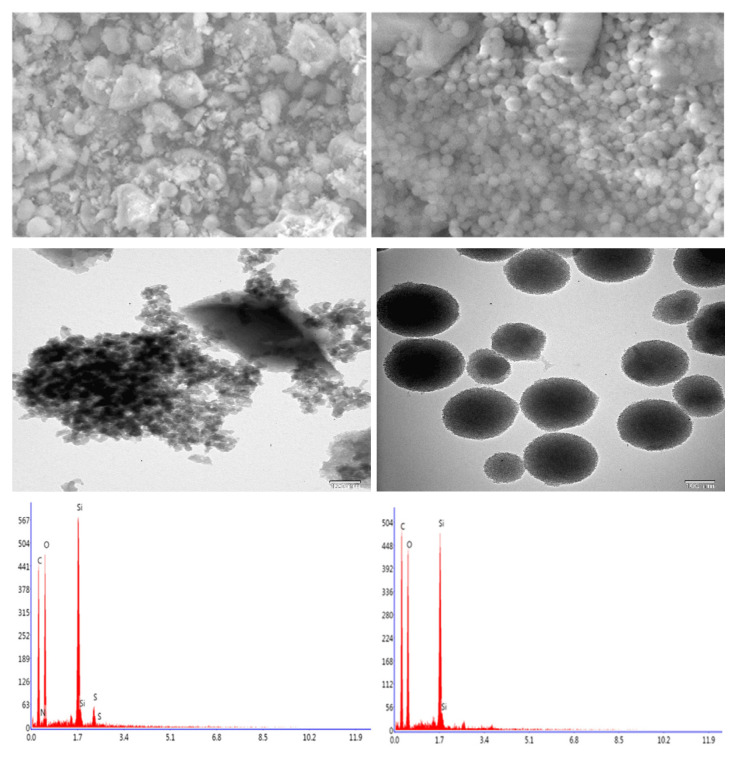
SEM and TEM images, and EDS analyses of MON (left) and PMO (right).

SEM and TEM analyses were carried out to obtain information about the morphological appearance of the obtained nanocarriers MON and PMO. SEM and TEM micrographs of the MON and PMO nanocarriers are given in Figure 3. SEM and TEM micrographs show that the MON structure was present in the form of a small number of regular structures and mostly as aggregates. For PMO, a more uniform and orderly structure was observed.

**Figure 4:**
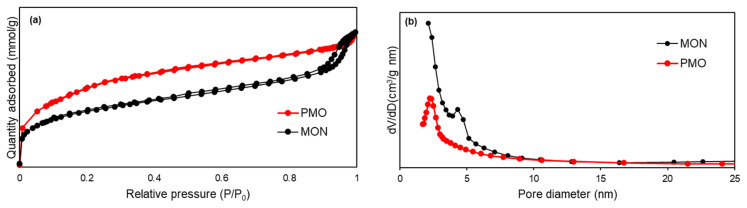
(a) N_2_ adsorption-desorption isotherm and (b) pore size distribution of MON and PMO.

Absorption-desorption isotherms and pore size distribution curves for MON and PMO are given in Figure 4. The Barrett–Joyner–Halenda (BJH) method was used to calculate the pore size distribution and the average pore diameter. The Brunauer–Emmett–Teller (BET) surface area (*S_BET_*) for MON was 828 m^2^/g, the total pore volume (*V_total_*) was 0.69 cm^3^/g, and the average pore diameter was 5.4 nm. For PMO, the BET surface area was calculated as 1215 m^2^/g, the *V_total_* was 0.82 cm^3^/g, and the average pore diameter was 3.4 nm.

**Figure 5:**
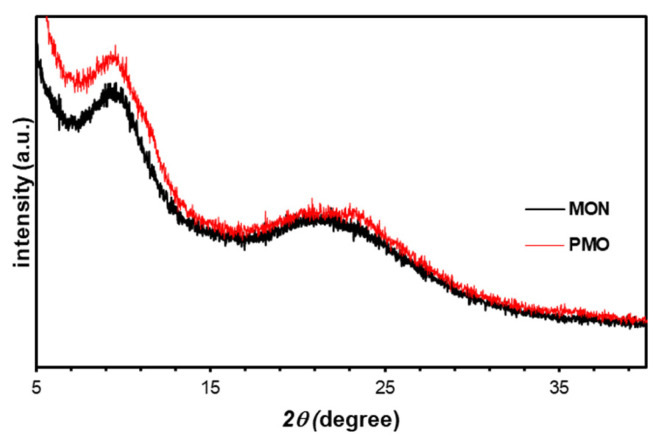
Wide-angle X-ray diffraction (XRD) patterns of MON and PMO.

The wide-angle powder X-ray diffraction patterns for MON and PMO are shown in Figure 5. According to the literature, the molecular size of BTSB is 7.6 Å, and after the use of BTSB with this size in the synthesis of MON and PMO, the formation of molecularly aligned bridges in the resulting nanomaterials can be characterized by XRD [39,41]. As a result of the molecular periodicity created by these structures, it has been reported that reflection signals, which are multiples of each other, should be observed in the wide-angle powder X-ray diffraction pattern [39,41]. Observation of reflection signals at 2θ = 10° and 20° in the resulting wide-angle powder pattern confirms that there is molecular periodicity in the MON and PMO structure.

**Figure 6:**
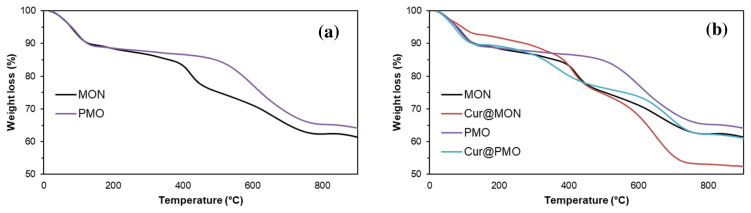
TGA curves of (a) MON and PMO, and (b) MON, Cur@MON, PMO, and Cur@PMO.

Finally, TGA analyses of MON and PMO were performed to calculate the organic content of the nanocarriers and are shown in Figure 6a. The 1% mass loss observed in the TGA curve below 150 °C is due to the solvents trapped and physically bound in the pores of MON and PMO. The mass loss for MON and PMO between 150 °C and 800 °C was recorded as 29% and 23%, respectively. These mass loss amounts can be attributed to the decomposition of the organic parts in the structure of MON and PMO due to temperature.

### 3.2. In vitro curcumin release from Cur@MON and Cur@PMO

**Scheme 2:**
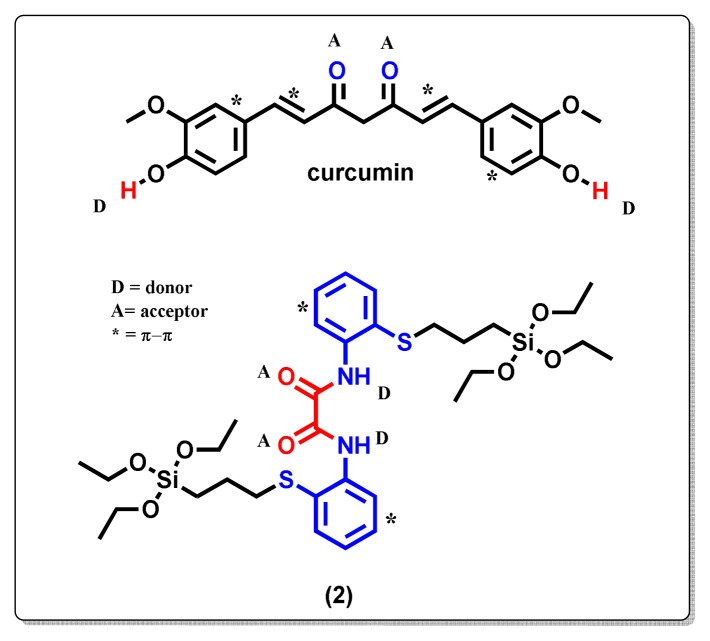
Possible interactions between curcumin and organosilane (2).

Hydrophobic curcumin was chosen as a model compound to determine the loading capacity of MON and PMO. With the help of equation 1 and TGA analyses, the loading capacities were calculated as 11.4% for MON and 2.9% for PMO (Figure 6b). As shown by the studies in the literature, the loading and release properties of the carrier change depending on the increase in the number and strength of the interactions between the carrier and the drug [1,2,6]. When we look at the drug loading mechanism between curcumin and MON, it can be thought that it originates from the intermolecular interactions with the organic groups included in the MON structure and the groups already present in the curcumin structure. Intermolecular interactions, mainly due to H bonds originating from H donors and acceptors present in both structures, and π–π interactions resulting from the presence of π-conjugated groups, play an important role in the interaction between curcumin and nanocarrier MON [40]. As a result of these interactions, it was observed that the amount of curcumin loaded on the MON carrier was higher than that on the PMO carrier. The possible types of interactions that may occur between the organosilane compound (2) and curcumin are shown in Scheme 2.

**Figure 7:**
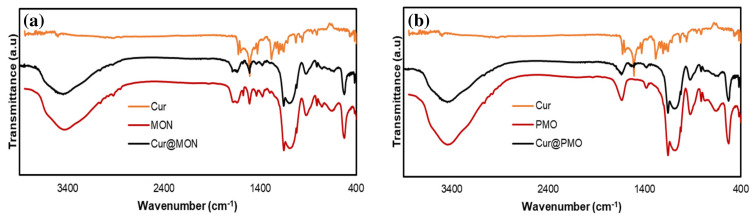
FT-IR spectra of curcumin, MON, and Cur@MON, and of curcumin, PMO, and Cur@PMO.

The overlaid FT-IR spectra of curcumin, MON, PMO, Cur@MON, and Cur@PMO are shown in Figure 7. It was seen in the FT-IR spectra that the fundamental vibrations in the FT-IR spectrum of MON were clearly evident in the FT-IR spectrum of curcumin-loaded Cur@MON. In particular, the stretching vibrations of the Si-O group in the network of the nanocarrier are clearly observed in the range of 1090–1140 cm^−1^. The presence of the C=O group of the oxamide in MON and Cur@MON was proved by the appearance of the stretching vibration at around 1640 cm^−1^. In the FT-IR spectrum of Cur@MON, the presence of weak vibrations observed in the region above 3400 cm^−1^ and in the region between 1800–2000 cm^−1^ confirms that curcumin was loaded into the MON structure. This was also confirmed for the curcumin-loaded Cur@PMO nanocarrier by observation of similar signals in its FT-IR spectrum.

**Figure 8:**
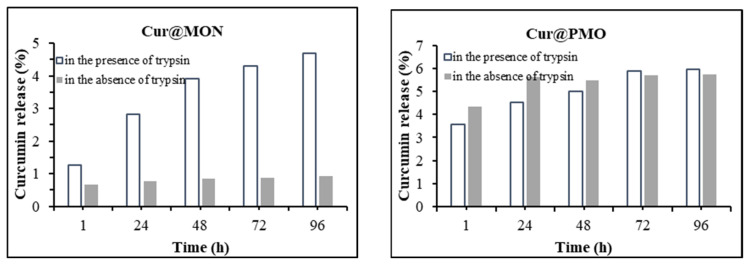
Curcumin release profiles of Cur@MON and Cur@PMO in the presence and absence of trypsin at pH = 7.4.

As a next step, an attempt was made to determine whether Cur@MON released curcumin at physiological pH = 7.4 and endosomal pH = 5.5. For this, Cur@MON was dispersed in PBS (pH = 7.4 and 5.5) containing 10% Tween-80 and mixed at 100 rpm at 37 °C for 4 days. Samples were taken from the release medium at regular intervals and centrifuged, and the absorbance of the supernatant was read at 420 nm. The amount of curcumin corresponding to the reading was calculated using the standard calibration curve. The graph of the results obtained from the 4-day measurements is given in Figure 8. The reference nanocarrier Cur@PMO was also subjected to the same experiment under the same conditions. The results showed that less than 1% of curcumin leaked from Cur@MON at pH = 7.4 and pH = 5.5. The low curcumin leakage rate proves that the drug-loaded Cur@MON structure has the potential to be used as a drug delivery system without using any pore-closing agent, and that the MON structure has a strong interaction with curcumin. For the reference material Cur@PMO, the leakage values of curcumin at pH = 5.5 at the end of the 1st day and the 4th day were approximately close to each other and around 5.5%. Although an increase in the leakage values for Cur@PMO was observed every day at pH = 7.4, it was observed that the value remained approximately constant at the end of the 4th day and reached 4.5%. These results revealed that MON leaked less loaded curcumin compared to PMO at pH = 7.4 and 5.5. These differences in results can be attributed to the fact that MON, unlike PMO, contains an organic group with an oxamide bridge in the silica network. It can also be said that the amide group of compound (2), located in the silica network of the MON structure, causes a stronger interaction between curcumin and the silica network, and, as a result, prevents curcumin from leaking through the nanocarrier.

To evaluate the effect of trypsin on curcumin release, Cur@MON and PBS (pH = 7.4) were placed in a test tube, and a sonicator was used to disperse Cur@MON in PBS. Then, the resulting release medium was allowed to mix at 100 rpm at 37 °C. Samples were taken from the release medium at regular intervals, and the absorbance of the supernatant was read on a UV-Vis spectrophotometer at a wavelength of 420 nm. The absorbance value was placed on the standard calibration curve, and the amount of curcumin released was calculated. Figure 8 shows the percentage of released curcumin versus time. It was observed that less than 1% was released from Cur@MON in the absence of trypsin protein at pH = 7.4, while nearly 5% of curcumin was released at the same pH in the presence of trypsin. For Cur@PMO, the curcumin release profile in the presence of trypsin was quite similar to that in the absence of trypsin, and no increase in curcumin release was observed. These results show that trypsin triggered the release of curcumin from Cur@MON, but had no effect on the release of curcumin from Cur@PMO. The expectation that the oxamide groups used in the MON structure would undergo partial degradation in the presence of trypsin protein and increase the release of curcumin is supported by these results.

## 4. Conclusions

Within the scope of this study, an oxamide-bridged mesoporous organosilica nanocarrier, which can be triggered in the presence of trypsin enzyme, was synthesized, and its drug loading and release properties were investigated. By adding the oxamide bridge compound (2) into the pore walls of the organosilica material, MON gained the ability to mimic enzymatic bond cleavage. Trypsin was used as a model enzyme to initiate the enzymatic bond cleavage of MON. After loading curcumin into MON, less than 1% leakage was observed at pH = 7.4. The fact that limited leakage occurs at physiological pH without using pore-closing agents indicates that there is a sufficiently strong interaction between the organic groups in the MON structure and curcumin to prevent this leakage. Curcumin release experiments at physiological pH showed that the release of curcumin from MON increased and partially occurred in the presence of trypsin compared to the environment without trypsin. It is a desired feature for a nanocarrier that the drug-loaded system can transport the drug without leaking, and release it at the desired place and time. To increase trypsin-responsive curcumin release rates, our studies on this subject are continuing with different approaches.

## Supplementary information

Figure S1 ^1^H-NMR spectrum of 2.

Figure S2 ^13^C-NMR (APT) spectrum of 2.

Figure S3 MALDI-TOF spectrum of 2.

Figure S4 UV-Vis spectrum of curcumin in ethanol.

Figure S5 Standard calibration curve of curcumin in concentration ranges from 0.625 μg/mL to 20 μg/mL ethanol.
