# Supplementary figures and images for: Synthesis, characterization, and drug release properties of enzyme-responsive oxamide-bridged mesoporous organosilica nanoparticles
Source: Turk J Chem. 2025 Apr 8;49(4):439–49. doi: 10.55730/1300-0527.3742 (PMC13052436; doi:10.55730/1300-0527.3742)

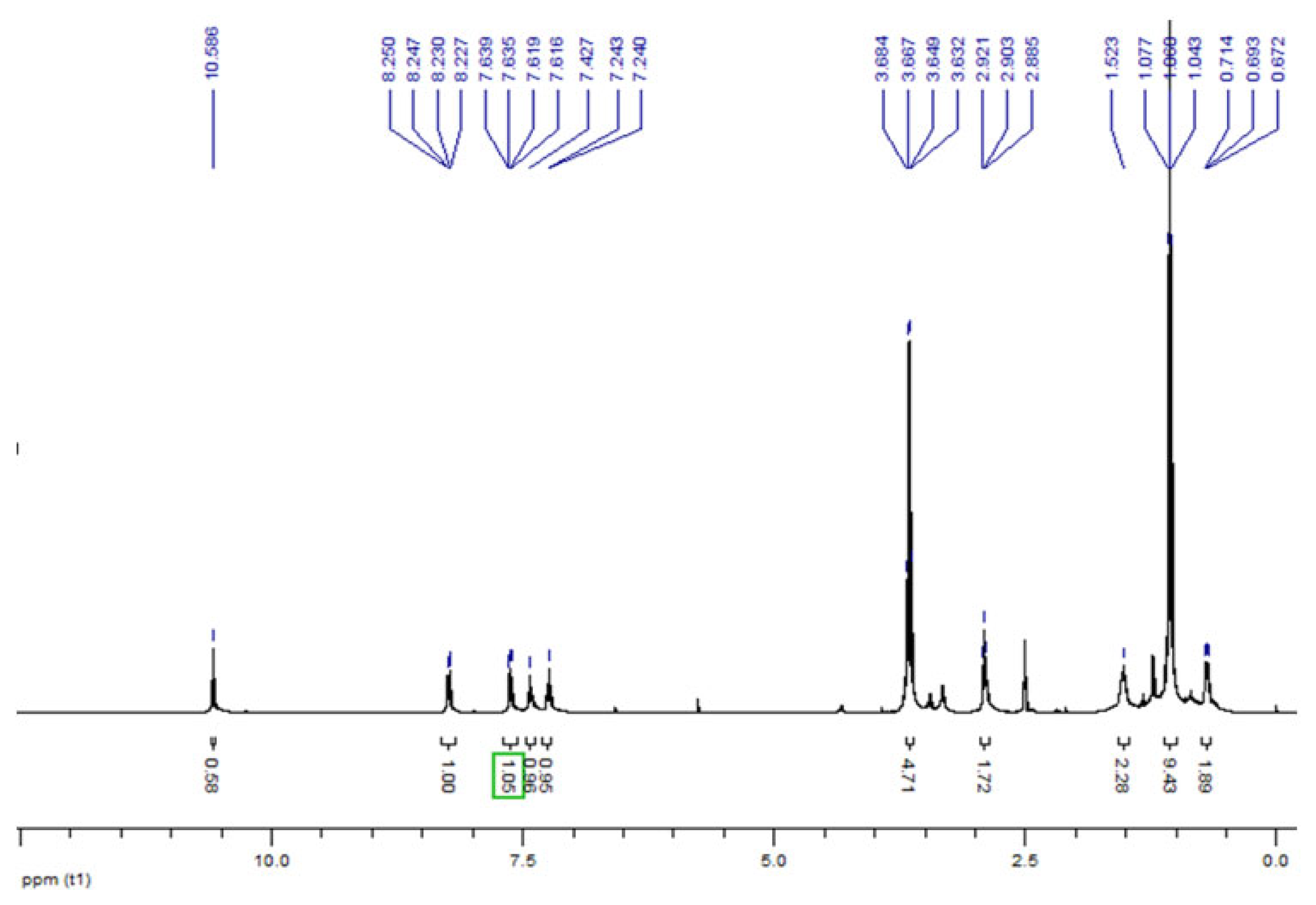

Supplement: Figure S1 — 1H-NMR spectrum of 2. [file tjc-49-04-439s1.tif]

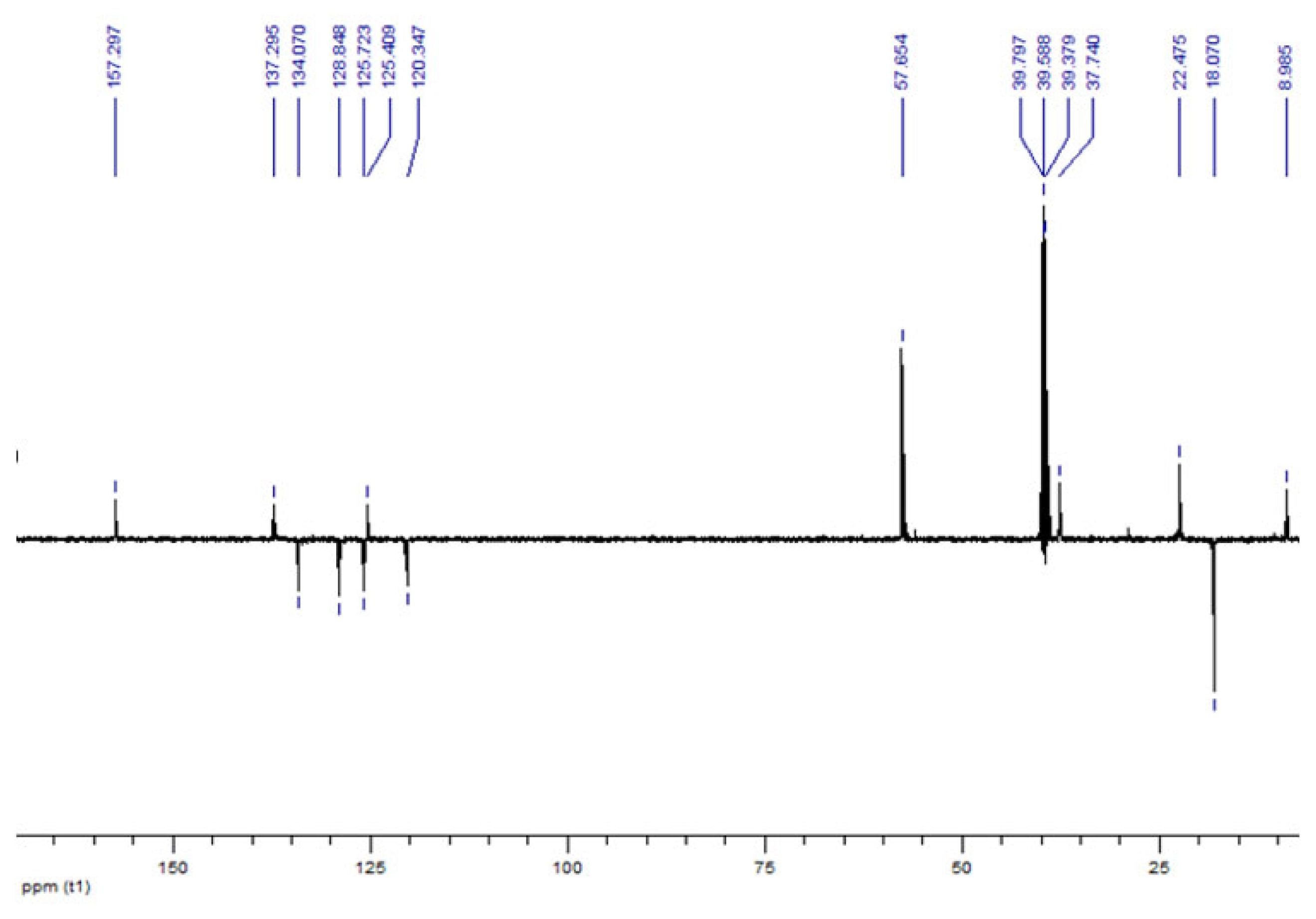

Supplement: Figure S2 — 13C-NMR (APT) spectrum of 2. [file tjc-49-04-439s2.tif]

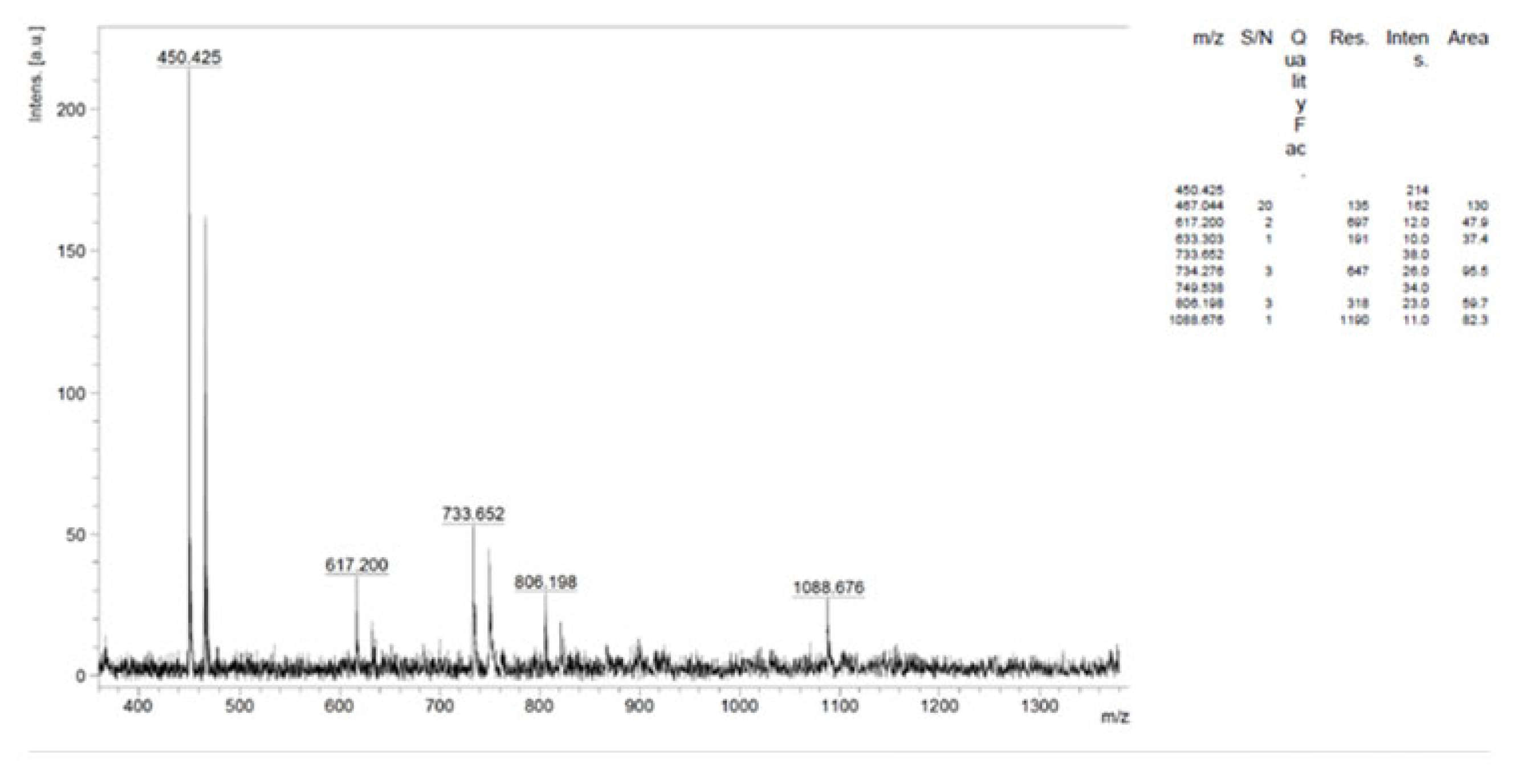

Supplement: Figure S3 — MALDI-TOF spectrum of 2. [file tjc-49-04-439s3.tif]

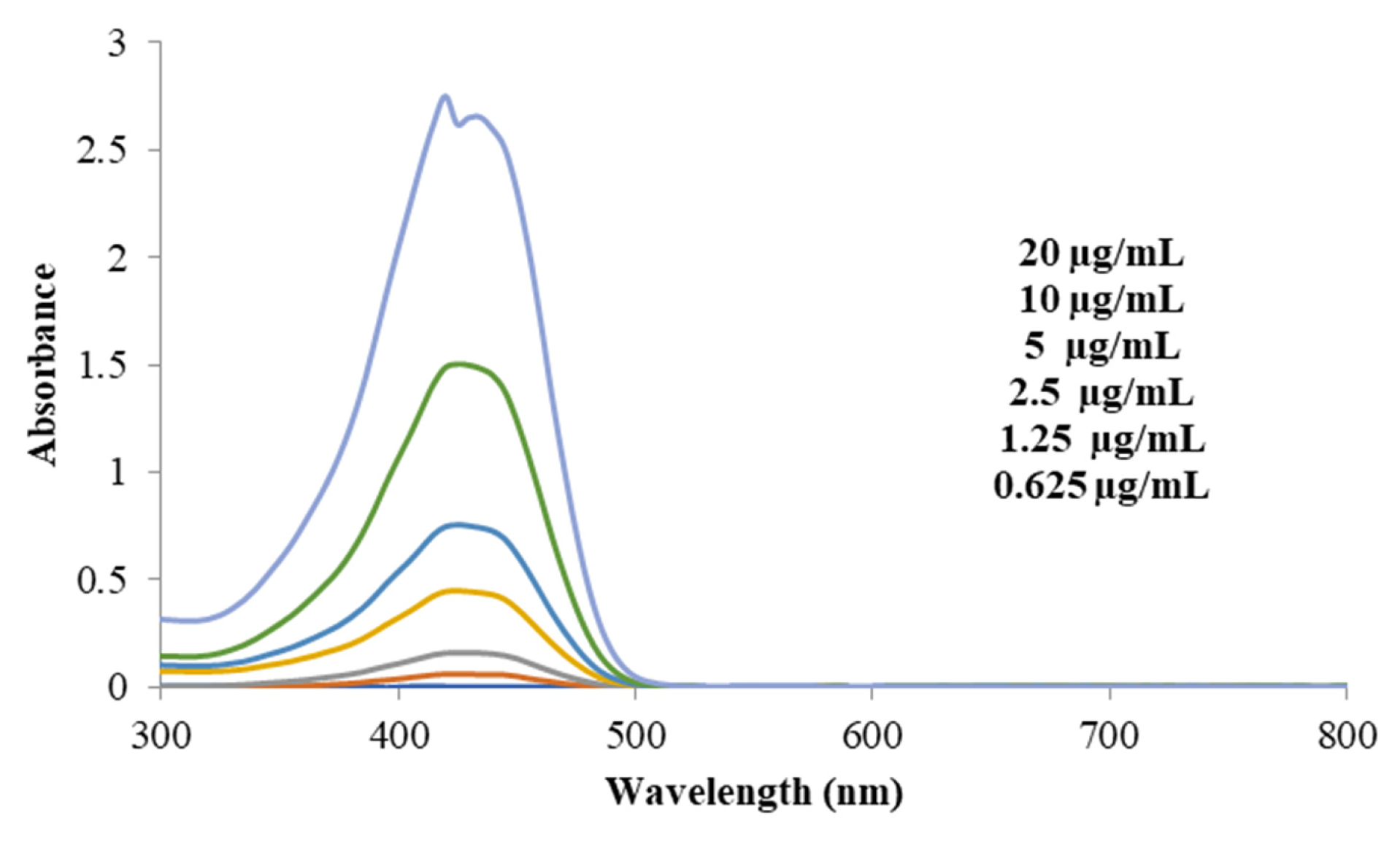

Supplement: Figure S4 — UV-Vis spectrum of curcumin in ethanol. [file tjc-49-04-439s4.tif]

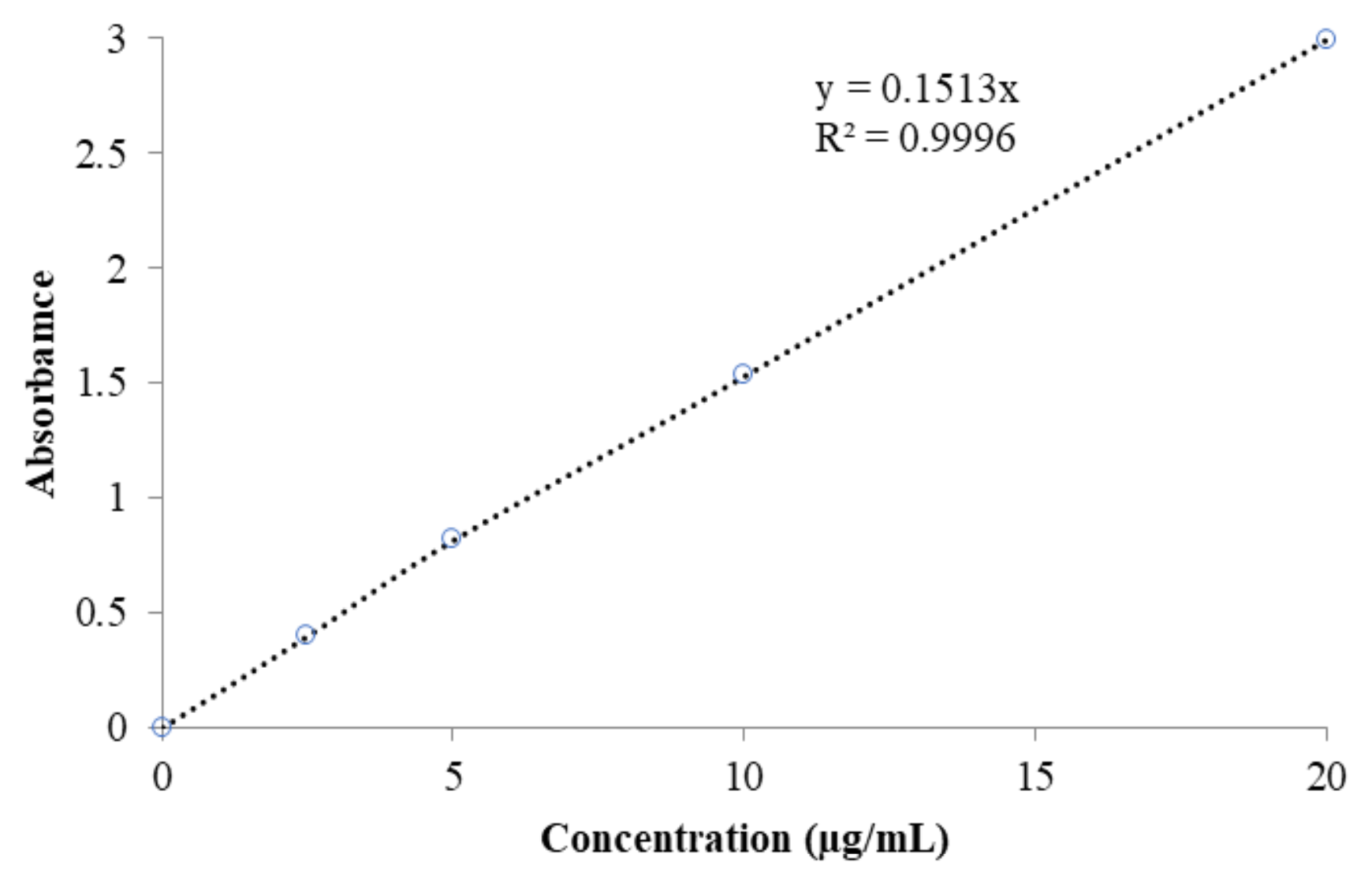

Supplement: Figure S5 — Standard calibration curve of curcumin in concentration ranges from 0.625 μg/mL to 20 μg/mL ethanol. [file tjc-49-04-439s5.tif]
